# Age-Related Differences in Prestimulus EEG Affect ERPs and Behaviour in the Equiprobable Go/NoGo Task

**DOI:** 10.3390/brainsci14090868

**Published:** 2024-08-28

**Authors:** Robert J. Barry, Frances M. De Blasio, Adam R. Clarke, Alexander T. Duda, Beckett S. Munford

**Affiliations:** Brain & Behaviour Research Institute, School of Psychology, University of Wollongong, Wollongong, NSW 2522, Australia; francesd@uow.edu.au (F.M.D.B.); aclarke@uow.edu.au (A.R.C.); aduda@uow.edu.au (A.T.D.); rm798@uowmail.edu.au (B.S.M.)

**Keywords:** prestimulus EEG components, poststimulus ERP components, behaviour, temporal and frequency PCA, developmental brain dynamics

## Abstract

Detailed studies of the equiprobable auditory Go/NoGo task have allowed for the development of a sequential-processing model of the perceptual and cognitive processes involved. These processes are reflected in various components differentiating the Go and NoGo event-related potentials (ERPs). It has long been established that electroencephalography (EEG) changes through normal lifespan development. It is also known that ERPs and behaviour in the equiprobable auditory Go/NoGo task change from children to young adults, and again in older adults. Here, we provide a novel examination of links between in-task prestimulus EEG, poststimulus ERPs, and behaviour in three gender-matched groups: children (8–12 years), young adults (18–24 years), and older adults (59–74 years). We used a frequency Principal Component Analysis (f-PCA) to estimate prestimulus EEG components and a temporal Principal Component Analysis (t-PCA) to separately estimate poststimulus ERP Go and NoGo components in each age group to avoid misallocation of variance. The links between EEG components, ERP components, and behavioural measures differed markedly between the groups. The young adults performed best and accomplished this with the simplest EEG–ERP–behaviour brain dynamics pattern. The children performed worst, and this was reflected in the most complex brain dynamics pattern. The older adults showed some reduction in performance, reflected in an EEG–ERP–behaviour pattern with intermediate complexity between those of the children and young adults. These novel brain dynamics patterns hold promise for future developmental research.

## 1. Introduction

The equiprobable Go/NoGo task is an interesting paradigm that bridges the active auditory Oddball and Go/NoGo tasks, which are traditional two-choice reaction time tasks. In the former, the target stimulus is presented with low probability (commonly 20%)—making it the unusual or “oddball” stimulus. In the latter, the target stimulus has high probability (commonly 80%)—making its response prepotent and requiring its inhibition for the correct withholding of a response to the rarer NoGo stimulus. The equiprobable task presents each stimulus at 50% probability and reduces the demand for active inhibition. It also benefits exploration of the competing Go/NoGo processing chains by providing equal numbers of stimulus–response pairings. As a simple two-choice reaction time task, it can be used with participants of a wide range of ages across the lifespan [1].

**Figure 1:**
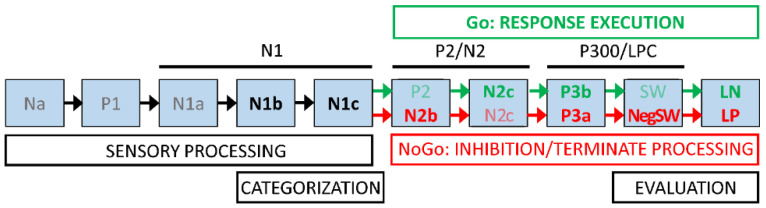
A processing schema for the equiprobable auditory Go/NoGo task. Sensory processing of the auditory stimulus is marked by the early P1 and N1 subcomponents as information is extracted to identify it as Go or NoGo. Categorisation is marked by P2/N2 components, beginning separate processing chains for Go (marked by P3b and the RT response) or NoGo (marked by P3a and termination of processing). Both chains may be followed by one or more slow waves (SWs) indicating evaluation of the stimulus/response pairing. Component labels are faded to indicate those not always seen. LPC = late positive complex; NegSW = negative SW; LN = late negativity; LP = late positivity.

With neurotypical adults, Barry and De Blasio [2] developed a processing schema linking event-related potential (ERP) components, derived via temporal Principal Component Analysis (t-PCA), to the perceptual and cognitive processing involved in completing the equiprobable auditory Go/NoGo task. In general, the initial stimulus evaluation processing stages (marked by the early P1/N1 components) are similar in both the Go and NoGo processing chains. When the stimuli are identified as Go or NoGo, marked by P2/N2 components, the processing chains diverge, as the Go imperative initiates the active response (marked by P3b) and the NoGo processing (marked by P3a) winds down without active responding. Subsequent components may reflect evaluative processing of that decision. This general schema has now been confirmed in young and older adults [1] and explored in children [3,4]. It has also been explored in relation to development between children and adults [5] and in younger and older children [6]. A current version of the schema is shown in Figure 1.

The development of an EEG over the lifespan has been well documented, with largely consistent results regardless of measure (power or amplitude). This is characterised by a reduction in slow wave activity below 10 Hz (delta, theta, and low alpha), with an increase in faster waveforms (high alpha and beta) from childhood to adulthood [7,8,9,10,11,12]. Healthy older adults generally show reduced delta and theta, some reduction in alpha, and increased beta activity [13,14,15,16]. These studies have explored development in the resting state (with eyes open and/or closed), without any task involved. We have previously noted that the EEG power/frequency distribution differs from rest to task. Karamacoska et al. [17,18] reported that the band powers in delta, theta, alpha, and beta all increased in young adults from the resting, eyes-open condition to prestimulus activity during the equiprobable Go/NoGo task, although we are unaware of similar investigations in child or older adult populations. We are also unaware of developmental information on EEG *during* the Go/NoGo or similar cognitive tasks across the lifespan. However, we have observed age-related global reductions in prestimulus delta and theta [19], as well as focal reductions in alpha and increases in beta [20] in healthy older adults compared with young adults within the equiprobable auditory Go/NoGo task, broadly consistent with the changes reported in the resting state.

In this study, we expand upon our earlier work by examining ERP components and behavioural efficiency from a developmental perspective across three groups: neurotypical children, young adults, and older adults. The novel aspect of this study is our investigation of the relationships between the ongoing intrinsic EEG activity present at stimulus onset and the subsequent ERP components observed in the task. Genesis of the early exogenous ERP components has been related to phase locking in the ongoing EEG, while the later endogenous components have been related to differential evoked brain activity in response to Go vs. NoGo stimuli [21]. These generative processes indicate the important role that EEG in the immediate prestimulus period plays in determining the ERP components and task performance. We examine EEG from the relatively quiet prestimulus period in the equiprobable Go/NoGo task, avoiding contamination from the major event-related changes occurring in the poststimulus period. We add to the novelty of this study by applying a data-driven approach to EEG measurement, using a frequency Principal Components Analysis (f-PCA) to extract the natural frequency components [15]. f-PCA is a simple extension of t-PCA, in which the ERP waveform (voltage vs. time) is replaced by the EEG frequency spectrum (voltage vs. frequency). The other parameters (electrodes, conditions, and participants) are identical. The data sets involved are analogous, differing only in variable labels; these labels do not impact the mathematics involved, and the PCA methods are identical. f-PCA is implemented here with improvements from our earlier proof-of-concept study within the brain dynamics context [22]. The extracted components are data-driven and avoid issues of arbitrary frequency band limits common in the literature [15].

The current exploratory study maps the links between the prestimulus EEG activity immediately before Go and NoGo stimulus onset, the Go and NoGo ERP components subsequently occurring, and the associated behavioural outcomes within this task. We examine these linkages in three groups, children, young adults, and older adults, to obtain a first look at developmental changes across the lifespan in the brain dynamics involved in this cognitive task.

## 2. Materials and Methods

### 2.1. Participants

The current study assessed 60 self-reported healthy participants, drawn evenly from three age groups, child, young adult, and older adult; the group characteristics are reported in Table 1. The participants were initially recruited and participated voluntarily in separate studies, and their raw archival data were re-analysed here with a new processing pipeline that was applied consistently across all participants. Informed consent was obtained from each volunteer (adult samples) or their parent/guardian (child sample), and the protocols were in accordance with the Declaration of Helsinki and were approved by the joint University of Wollongong/South East Sydney and Illawarra Area Health Service Human Research Ethics Committee.

All participants self-reported their handedness, abstinence from caffeine and tobacco for a minimum of 2.5 h prior to their testing session, and their health status. Participants who reported prior head trauma resulting in unconsciousness, epileptic seizures, and prior or current psychiatric illness and/or psychoactive drug use were excluded, as were those found to have high error rates and those with high levels of EEG artefacts. In addition, the child sample was screened for learning disabilities and low IQ scores (<85), and the older adults were screened for possible cognitive impairment (Rowland Universal Dementia Assessment Scale [RUDAS] score ≤ 22; [23]). As seen in Table 1, care was taken to minimise confounds of biological sex and handedness, resulting in a final sample size of 20 participants per group.

### 2.2. Task and Procedure

Following informed consent and screening, participants were fitted with electrophysiological recording equipment and sat in a quiet and dimly lit testing room. Continuous EEG was recorded while participants completed a battery of tasks, some of which differed between the groups, although all participants completed at least two blocks of an equiprobable auditory Go/NoGo task, the task assessed in this study. The stimuli were 1000 Hz and 1500 Hz tones, each of 50 ms duration; these were inclusive of a 5 ms rise/fall and played at 60 dB SPL in the child and young adult samples but included a rise/fall of 15 ms and were played at 70 dB SPL in the older adult sample to ensure tone discriminability due to the link between age-related hearing decline and cognition [24,25]. All participants received the stimuli with a fixed onset asynchrony (SOA) of 1100 ms, and each of the assessed blocks contained 75 Go and 75 NoGo tones that were shuffled to obtain a random presentation order that varied between participants. The tone assigned as the Go stimulus was held consistent between the blocks and was counterbalanced across participants within the child and young adult samples; however, this designation alternated between the blocks within the older adult sample, and the tone assigned as the first Go stimulus was counterbalanced within this group. Participants were instructed to respond to the designated Go tone with a button-press response using their dominant hand, and brief breaks were offered between the blocks to minimise fatigue.

### 2.3. Electrophysiological Recording

For all participants, EEG data were recorded from 19 scalp sites (Fp1, Fp2, F7, F3, Fz, F4, F8, T7, C3, Cz, C4, T8, P7, P3, Pz, P4, P8, O1, and O2), and electro-ocular (EOG) data were recorded from four facial electrodes positioned above/below the left eye to capture vertical activity and beyond the outer canthus of each eye to capture horizontal activity. These data were recorded at 512 Hz via a 16-bit A/D system (AMLAB II) with a linked ear reference for the child (data from 0.03 to 35 Hz) and young adult (data from 0.15 to 30 Hz) groups. The older adult data (DC to 30 Hz) were recorded at 1000 Hz using a Neuroscan Synamps 2 amplifier with Neuroscan Acquire software (Compumedics, Version 4.3.1), with the left ear serving as the online reference and the right ear recorded as a separate channel. All electrodes were tin, and care was taken to keep impedances below 10 kΩ.

### 2.4. Data Quantification

All data processing was completed in MATLAB (The Mathworks; Version 9.7.0.1190202, R2019b), using EEGLAB (version 2022.0) [26], the ERP PCA Toolkit (version 2.891) [27], and custom scripts, with the processing pipeline applied uniformly across the groups unless otherwise specified.

The raw continuous EEG data for each participant were first bandpass-filtered (0.1–30 Hz, zero-phase shift) to ensure uniformity in their spectral range, and the older adult data were re-referenced to the digital equivalent of linked ears. Non-overlapping epochs were extracted at −500 to 600 ms relative to stimulus onset for valid Go and NoGo trials. Valid Go trials were those with a button-press response within the 100–600 ms poststimulus period, and valid NoGo trials were those with no response recorded within the 1100 ms SOA period following NoGo stimulus onset; these criteria were used to extract the error rates for behavioural analysis, with the total Go errors further subdivided into errors of omission (i.e., no response recorded within SOA period), fast RT errors (i.e., a Go trial with RT ≤ 100 ms), and slow RT errors (i.e., a Go trial with RT ≥ 600 ms, thus overlapping the prestimulus period of the subsequent trial).

The epochs were manually visualised in EEGLAB, and those with identifiable flatlines and dropouts in any of the four EOG channels were rejected. The Gratton et al. [28] eye movement correction procedure was then implemented using the MATLAB function gratton_emcp.m (retrieved from https://github.com/kylemath/MathewsonMatlabTools/blob/master/EEG_analysis/gratton_emcp.m; accessed on 21 December 2023). Following EOG correction, the epochs were again manually visualised; epochs with residual EOG artefacts were rejected, and poor-quality scalp channels were interpolated using EEGLAB’s spherical interpolation function. Although this may cause “ghosting” issues from rank reduction in an Independent Component Analysis (ICA) [29], it does not pose a problem for our PCAs here because we selected only components above a variance threshold of 1.5%. No more than 3 of the 19 channels (15.8%) were interpolated for any individual participant, and the interpolation data are summarised in Table 2 for each group. The EEG data for the older adults were then down-sampled to 512 Hz, to match the sample rate in the child and young groups.

The EOG-corrected epochs for all participants and groups were baseline-corrected (−100 to 0 ms), and a three-step automatic artefact rejection procedure was run similar to [30]; this process rejected epochs detected as having extreme amplitudes > 150 µV, voltage jumps > 50 µV between datapoints, and those with a maximum voltage change < 0.05 µV in any 100 ms period. Finally, the remaining trials with a button-press response within their prestimulus period were identified and rejected to remove contamination of the prestimulus spectra. To maintain the signal-to-noise ratio within subjects between conditions, the number of accepted epochs was matched between Go and NoGo. This number was set (within subject) by the condition with the lower number of accepted trials, and the corresponding number of trials was then randomly selected for analysis in the alternate condition. That is, if there were 61 Go and 68 NoGo epochs, all of the Go epochs were assessed and 61 epochs were randomly selected for analysis in NoGo. Table 2 presents a summary of the number of epochs assessed in this study for each group. From the assessed Go trials, the mean reaction time (RT) and RT variability (standard deviation in RT) data were extracted for each participant and stored with the Go and NoGo error rate data for later behavioural analysis.

To quantify the natural prestimulus spectral components, the prestimulus period (−500 to 0 ms) of each accepted epoch was extracted and DC-corrected, followed by the application of a 10% Hanning window. The windowed data were zero-padded to 512 points and submitted to Discrete Fourier Transformation (DFT), yielding single-sided prestimulus spectral amplitudes with 1 Hz resolution after corrections for the use of the Hanning window (correction factor of 1.05) and zero-padding (correction factor of 2). The DC-30 Hz within-subject mean spectra were then derived separately for Go and NoGo and submitted to f-PCA using the ERP PCA Toolkit; separate PCAs were conducted for each group (child, young, and older), and each included the prestimulus spectra for Go and NoGo given the equiprobable nature of the task and the observed consistency in the spectra between conditions. For each of the three PCAs, the input consisted of 760 cases (20 participants × 19 channels × 2 conditions) and 31 variables (DC-30 Hz at 1 Hz resolution), providing a case-to-variable ratio of 24.5. Following the recommendations in [15], PCAs were conducted with the covariance matrix, Kaiser normalisation, and unrestricted Promax rotation of 31 components. Frequency components accounting for ≥1.5% variance were considered for analysis, and the within-subject global mean peak amplitude was extracted across the Go and NoGo conditions for those identified.

To quantify the ERP components, the ERP period (−100 to 600 ms) was extracted from the EOG-corrected epochs identified for analysis, and the mean Go and NoGo ERPs were derived within subjects. The average ERPs were half-sampled to 256 Hz and submitted to separate t-PCAs for each group (child, young, and older) and condition (Go and NoGo). For each of the six PCAs, the input comprised 380 cases (20 participants × 19 channels × 1 condition) and 179 variables (−100 to 600 ms at 3.9 ms resolution), providing a case-to-variable ratio of 2.1. PCAs were conducted with the covariance matrix, Kaiser normalisation, and unrestricted Varimax rotation of 179 components following the recommendations in [31]. Temporal components accounting for ≥1.5% variance were considered for analysis, and the within-subject global mean peak amplitude was extracted separately for each Go and NoGo component identified.

### 2.5. Statistical Analysis

All analyses were undertaken in JASP (version 0.18.3) [32]. Spearman’s rank-order correlations (rho, ρ) were conducted separately for each group, and each assessed the relationships between the global mean peak amplitudes for each identified prestimulus f-PCA EEG component, t-PCA ERP component, and behavioural measures. All correlations had 18 degrees of freedom and were assessed with two-tailed significance. An alpha level of 0.05 was used to detect significant associations, and due to the exploratory nature of this investigation, near significant effects (0.05 < *p* ≤ 0.10) are also reported. The 95% confidence intervals (CIs) are reported for the Spearman correlations [33].

## 3. Results

### 3.1. Child Group

Behaviourally, the child group had an average of 6.3% commission errors in NoGo and a 13.5% total error rate in Go, which comprised 3.1% omission errors, 0.5% fast RT errors, and 9.9% slow RT errors. Their mean RT was 389 ms (*SD* = 38), and their RT variability was 85 ms (*SD* = 16).

**Figure 2:**
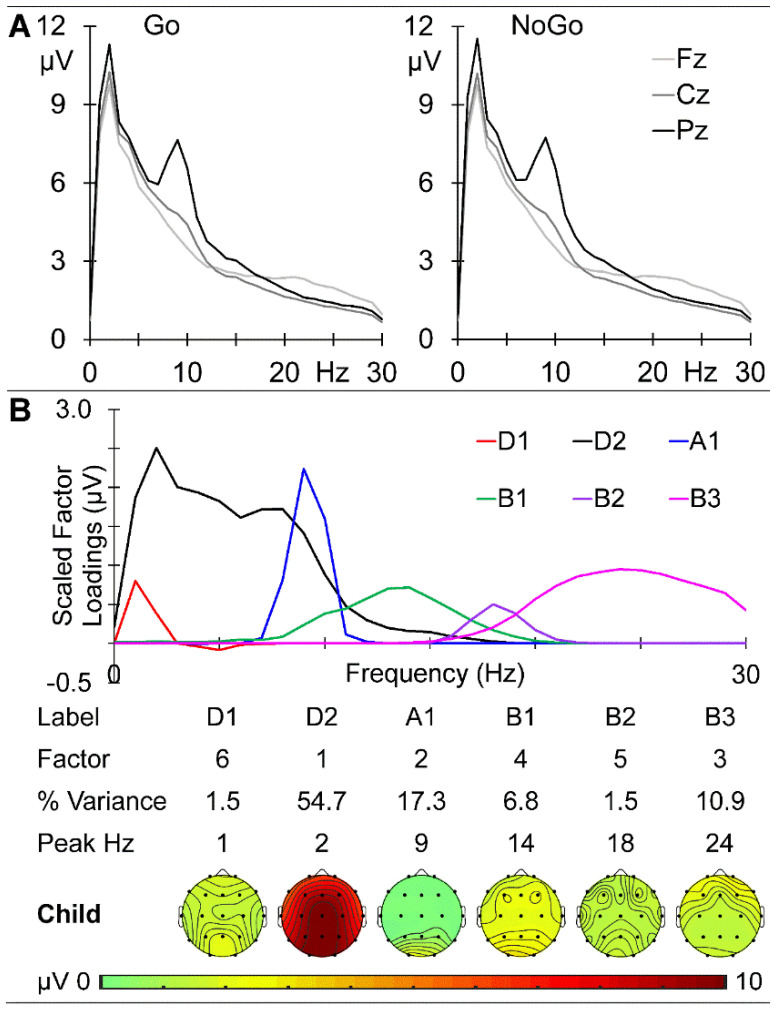
Child prestimulus spectra. (**A**) Amplitude as a function of frequency at the midline sites before Go (**left**) and NoGo (**right**) stimuli. (**B**) f-PCA outputs showing the scaled factor loadings as a function of frequency above the components in temporal order, together with the % variance carried and their peak frequency. Below this information is their topographic distribution with voltage scale.

The child group prestimulus EEG spectra at the midline sites (Fz, Cz, and Pz) are shown in Figure 2A. There is a clear parietal peak at 2 Hz in the delta band and a marked parietal peak at 9 Hz in the alpha band, with activity declining over the beta range and becoming frontal. The prestimulus activities before the Go and NoGo stimuli were very similar, with the absolute maximum amplitude difference across frequency and sites being 0.286 μV. These data were submitted to a combined f-PCA, which yielded two delta components (D1 at 1 Hz and D2 at 2 Hz), one alpha component (A1 at 9 Hz), and three beta components (B1 at 14 Hz, B2 at 18 Hz, and B3 at 24 Hz), each carrying at least 1.5% variance, together carrying 92.7% of the spectral variance. Their scaled factor loadings and topographical headmaps are shown in Figure 2B.

**Figure 3:**
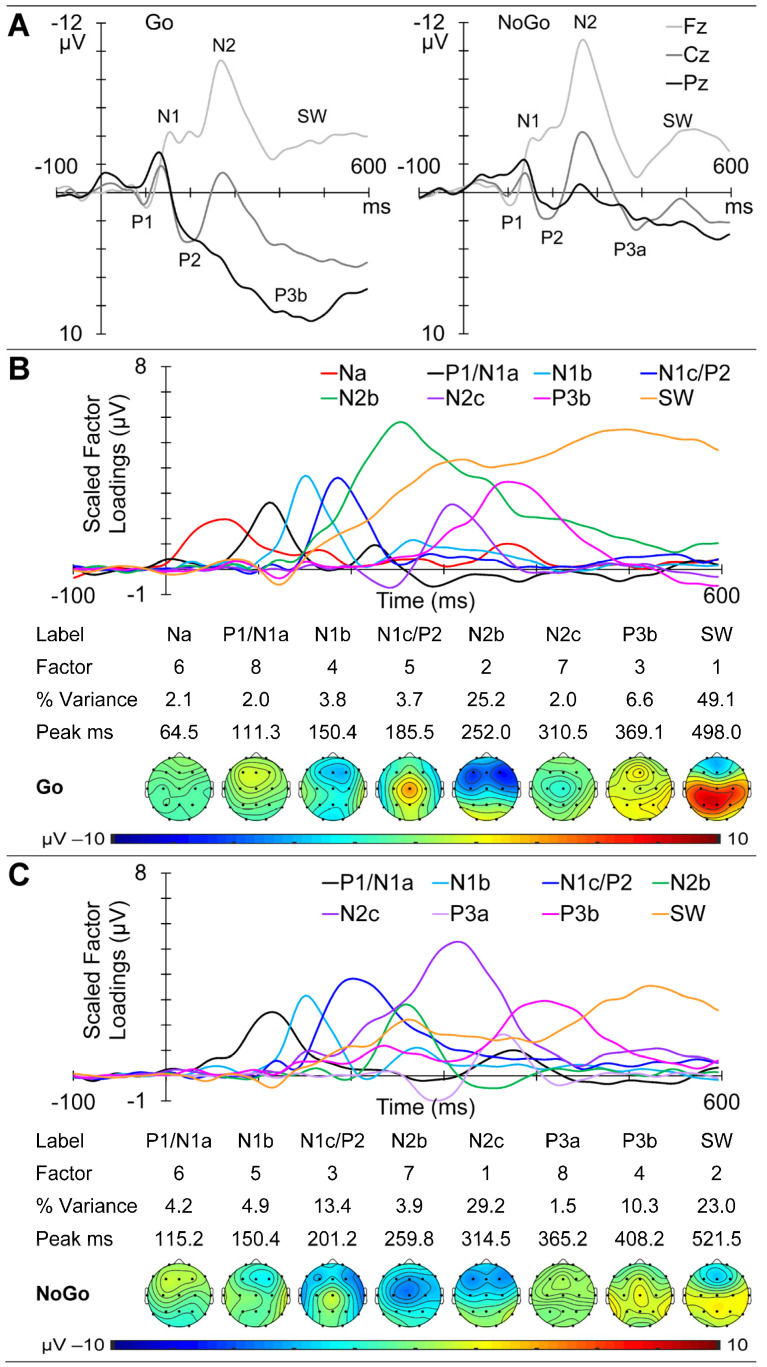
Child ERP results. (**A**) Morphology of the Go and NoGo responses at the midline sites as a function of time from stimulus onset. (**B**) t-PCA outcomes for Go: scaled factor loadings are shown above the component information and component headmaps. (**C**) NoGo component results.

GM ERPs from the child group are shown in Figure 3A, with Go ERPs at the midline sites (Fz, Cz, and Pz) on the left and NoGo on the right. Clear P1 and N1 components are apparent in both sets, followed by a large frontal N2, with marked morphological Go/NoGo differences most apparent from approximately 250 ms. The Go ERP f-PCA yielded eight components carrying more than 1.5% variance each, for a total of 94.5% of the ERP variance. These were identified as Na, P1/N1a, and N1b, followed by N1c/P2, N2b, N2c, P3b, and SW. Their scaled factor loadings, component information (variance and peak latency), and topographic headmaps are shown in Figure 3B. The NoGo ERP f-PCA also yielded eight components carrying at least 1.5% variance each, for 90.4% of the total variance. These were identified as a P1/N1a, N1b, and N1c/P2, followed by N2b, N2c, P3a, P3b, and SW. Their scaled factor loadings, component information (variance and peak latency), and topographic headmaps are shown in Figure 3C.

#### Child Global Amplitude Correlation Analyses

Spearman correlations indicated that Go Na was somewhat inversely affected by A1 (ρ = 0.41, *p* = 0.07, CI = [−0.06, 0.73]) and B1 (ρ = 0.42, *p* = 0.07, CI = [−0.05, 0.74]); that is, when the A1 and B1 amplitudes were larger, the Na amplitudes tended to be more positive (i.e., smaller). The global positivity of the Go P1/N1a component was significantly affected directly by D1 (ρ = 0.55, *p* = 0.01, CI = [0.11, 0.81]), inversely by B2 (ρ = −0.46, *p* = 0.04, CI = [−0.76, 0.00]), and somewhat affected inversely by B3 (ρ = −0.41, *p* = 0.08, CI = [−0.73, 0.06]). Go N1c/P2 positivity was inversely affected by D2 (ρ = −0.44, *p* = 0.05, CI = [−0.75, 0.02]) and A1 (ρ = −0.58, *p* = 0.01, CI = [−0.83, −0.14]) and somewhat by B1 (ρ = −0.39, *p* = 0.09, CI = [−0.72, 0.08]). Go N2b negativity was somewhat affected inversely by A1 (ρ = 0.43, *p* = 0.06, CI = [−0.03, 0.74]) and directly by B2 (ρ = −0.42, *p* = 0.07, CI = [−0.74, 0.05]). Go P3b positivity was inversely affected by D2 (ρ = −0.45, *p* = 0.05, CI = [−0.76, 0.01]), while Go SW positivity was directly affected by D2 (ρ = 0.45, *p* = 0.05, CI = [−0.01, 0.76]) and B1 (ρ = 0.58, *p* = 0.01, CI = [0.15, 0.83]). Negativity in the NoGo N1b was somewhat directly affected by B3 (ρ = −0.39, *p* = 0.09, CI = [−0.72, 0.08]), N2b was inversely affected by A1 (ρ = 0.65, *p* = 0.01, CI = [0.25, 0.86]), and N2c was directly affected by B2 (ρ = −0.48, *p* = 0.03, CI = [−0.77, −0.02]). Positivity in the NoGo P3b was somewhat directly affected by D2 (ρ = 0.41, *p* = 0.08, CI = [−0.06, 0.73]), while the SW positivity was inversely affected by D1 (ρ = −0.54, *p* = 0.02, CI = [−0.80, −0.09]) and directly by D2 (ρ = 0.55, *p* = 0.01, CI = [0.11, 0.81]).

Child behaviour measures showed six links with their prestimulus EEG components. Go RT variability was somewhat affected inversely by A1 (ρ = −0.41, *p* = 0.07, CI = [−0.73, 0.06]). Go omissions and Go total errors were somewhat affected directly by D2 (ρ = 0.42, *p* = 0.07, CI = [−0.05, 0.74] and ρ = 0.41, *p* = 0.07, CI = [−0.06, 0.73], respectively). NoGo commissions were significantly affected directly by D2 (ρ = 0.51, *p* = 0.02, CI = [0.06, 0.79]) and B3 (ρ = 0.45, *p* = 0.05, CI = [−0.01, 0.76]) and somewhat affected inversely by D1 (ρ = −0.42, *p* = 0.06, CI = [−0.74, 0.05]).

ERP–behaviour links were also apparent. Go RT variability was somewhat directly associated with Go N2b (ρ = −0.40, *p* = 0.08, CI = [−0.72, 0.07]) and inversely with Go N2c (ρ = 0.38, *p* = 0.10, CI = [−0.09, 0.71]) and Go P3b (ρ = −0.42, *p* = 0.07, CI = [−0.73, 0.05]). Go omission errors were inversely related to Go P3b (ρ = −0.59, *p* = 0.01, CI = [−0.83, −0.16]) and somewhat so to Go P1/N1a (ρ = −0.42, *p* = 0.07, CI = [−0.74, 0.05]). Slow RT errors and total Go errors were each inversely related to Go P3b (ρ = −0.52, *p* = 0.02, CI = [−0.79, −0.07] and ρ = −0.62, *p* = 0.01, CI = [−0.85, −0.21], respectively).

**Figure 4:**
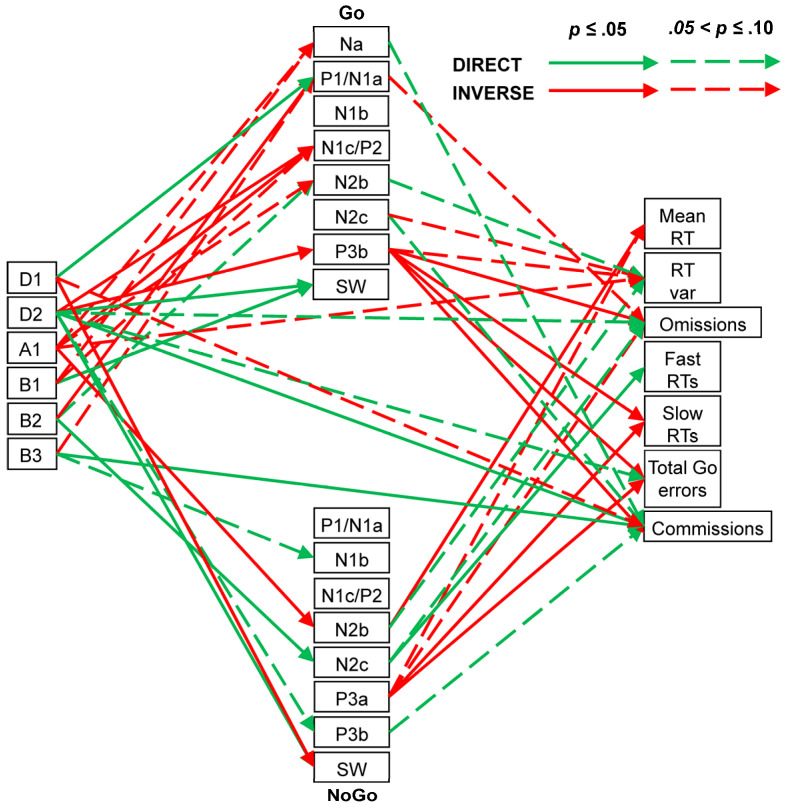
Child dynamics summary showing links between prestimulus frequency components (**left**), ERP components (centre: Go above NoGo), and behavioural measures (**right**).

Mean RT was inversely related to NoGo N2b negativity (ρ = 0.45, *p* = 0.05, CI = [−0.01, 0.76]) and somewhat inversely related to NoGo P3a positivity (ρ = −0.43, *p* = 0.06, CI = [−0.74, 0.04]), while RT variability was somewhat directly related to NoGo N2b negativity (ρ = −0.41, *p* = 0.07, CI = [−0.73, 0.06]). Go omissions were somewhat directly related to NoGo N2c negativity (ρ = −0.40, *p* = 0.08, CI = [−0.72, 0.07]) and inversely to P3a positivity (ρ = −0.40, *p* = 0.08, CI = [−0.72, 0.07]). Fast RT errors were directly related to NoGo N2c negativity (ρ = −0.51, *p* = 0.02, CI = [−0.79, −0.06]), while slow RT errors and Go total errors were inversely related to NoGo P3a (ρ = −0.50, *p* = 0.03, CI = [−0.78, −0.04] and ρ = −0.48, *p* = 0.03, CI = [−0.77, −0.02], respectively). NoGo commission errors were somewhat directly related to NoGo P3b (ρ = 0.39, *p* = 0.09, CI = [−0.08, 0.72]) and showed a significant inverse association with Go P3b (ρ = −0.54, *p* = 0.01, CI = [−0.80, −0.09]) and near-significant direct associations with the negativity in Go Na (ρ = −0.40, *p* = 0.08, CI = [−0.73, 0.07]) and Go N2c (ρ = −0.44, *p* = 0.05, CI = [−0.75, 0.03]). These links are shown in Figure 4.

### 3.2. Young Adult Group

The young group averaged 1.3% NoGo commission errors and a total Go error rate of 6.9%, reflecting 2.1% Go omission errors, 0.1% fast RT errors, and 4.6% slow RT errors. Their mean RT was 352 ms (*SD* = 57), and their RT variability was 71 ms (*SD* = 13).

**Figure 5:**
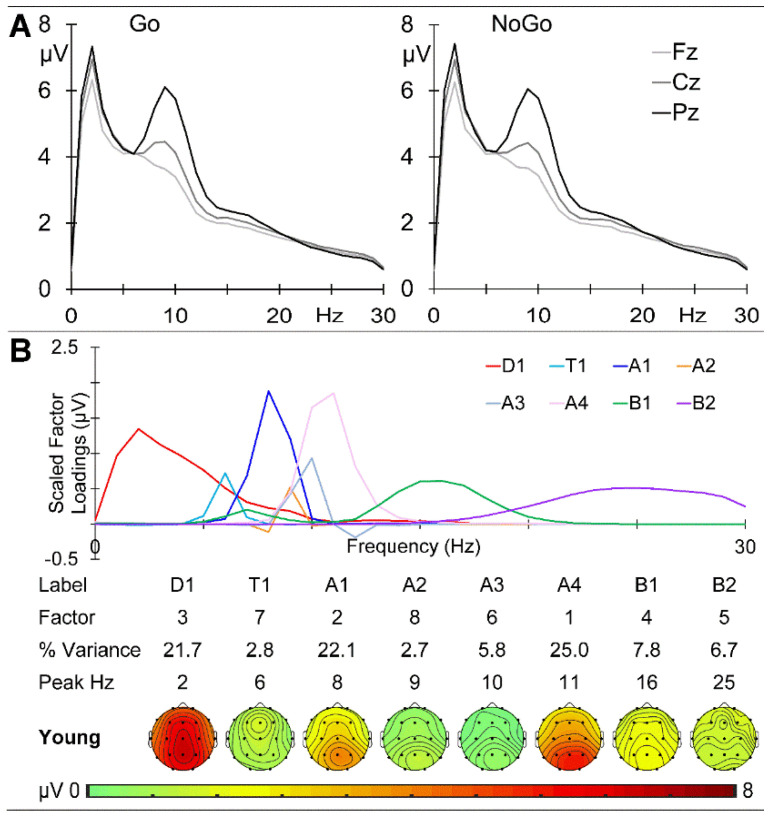
Young adult prestimulus spectra. (**A**) Amplitude at the midline sites before Go (**left**) and NoGo (**right**) stimuli. (**B**) f-PCA outputs with the scaled factor loadings above the components in temporal order, together with their % variance, peak frequency, and topographic headmaps.

The GM young group prestimulus EEG spectra at the midline sites are shown in Figure 5A. There is a clear centroparietal peak at 2 Hz in the delta band and a marked parietal peak at 9 Hz in the alpha band, with activity declining over the beta range. The prestimulus activities before the Go and NoGo stimuli were very similar, with the absolute maximum amplitude difference across sites and frequency being 0.203 μV. A combined f-PCA yielded one delta component (D1 at 2 Hz), one theta component (T1 at 6 Hz), four alpha components (A1 at 8 Hz, A2 at 9 Hz, A3 at 10 Hz, and A4 at 11 Hz), and two beta components (B1 at 16 Hz and B2 at 25 Hz), each carrying at least 1.5% variance and together carrying 94.6% of the spectral variance. Their scaled factor loadings and topographic headmaps are shown in Figure 5B.

**Figure 6:**
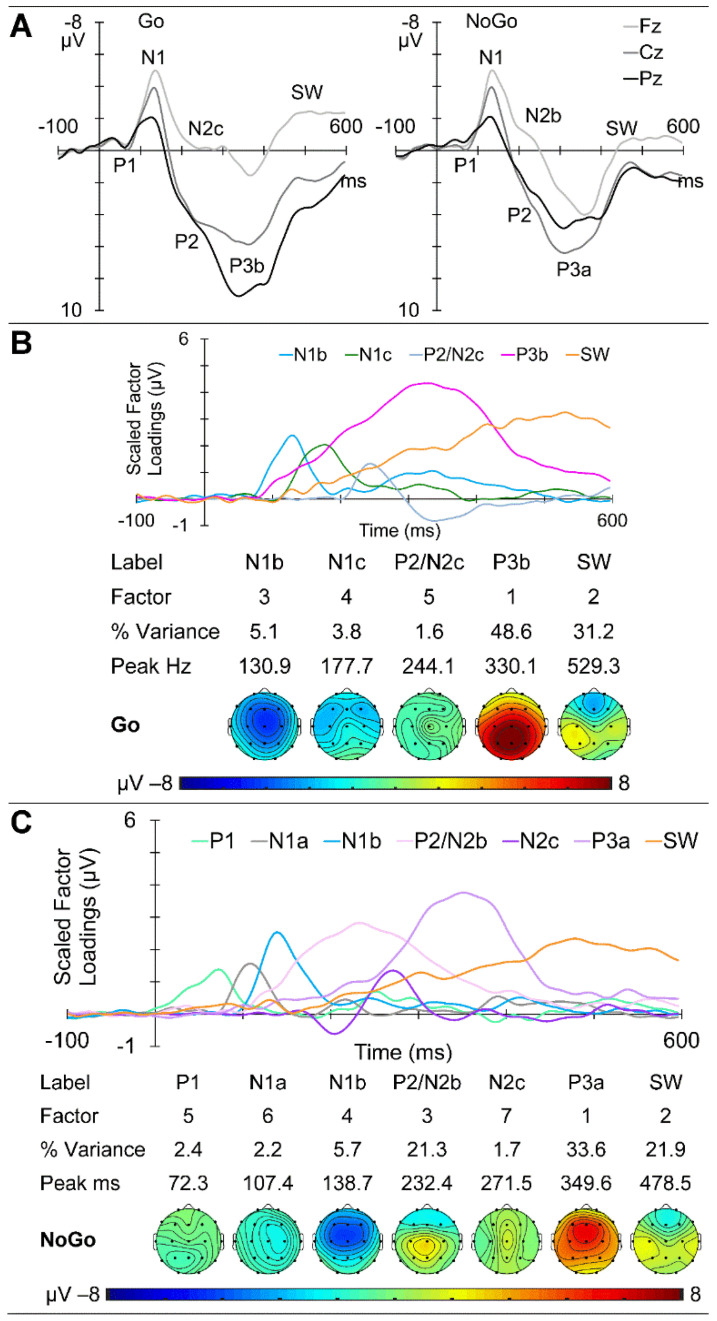
Young adult ERP results. (**A**) Go and NoGo response morphology at the midline sites as a function of time from stimulus onset. (**B**) t-PCA outcomes for Go: scaled factor loadings above the component information and component headmaps. (**C**) NoGo component results.

GM ERPs from the young group are shown in Figure 6A, with Go ERPs at the midline sites on the left and NoGo on the right. Clear P1 and N1 components are apparent in both sets, with a larger Go P1 and later NoGo N1. Subsequent Go/NoGo morphology differences are apparent from approximately 150 ms. The Go ERPs yielded five components carrying more than 1.5% variance each, for a total of 90.3% of the ERP variance. These were identified as N1b, followed by N1c, P2/N2c, P3b, and SW. Their scaled factor loadings, component information, and topographic headmaps are shown in Figure 6B. The NoGo ERPs yielded seven components carrying more than 1.5% variance each, for a total of 88.8% of the overall variance. These were identified as a P1, N1a, and N1b, followed by a P2/N2b, N2c, P3a, and SW. Their scaled factor loadings, component information, and topographic headmaps are shown in Figure 6C.

#### Young Global Amplitude Correlation Analyses

In the young group, Spearman correlations indicated that Go P2/N2c positivity was somewhat inversely affected by A4 (ρ = −0.42, *p* = 0.07, CI = [−0.74, 0.05]), while Go SW positivity was directly affected by D1 (ρ = 0.45, *p* = 0.05, CI = [−0.01, 0.75]) and somewhat affected by A1 (ρ = 0.39, *p* = 0.09, CI = [−0.08, 0.72]). NoGo P1 was inversely affected by T1 (ρ = −0.40, *p* = 0.08, CI = [−0.73, 0.07]), while NoGo P2/N2b positivity was directly affected by A2 (ρ = 0.47, *p* = 0.04, CI = [0.01, 0.77]) and A3 (ρ = 0.44, *p* = 0.05, CI = [−0.02, 0.75]).

**Figure 7:**
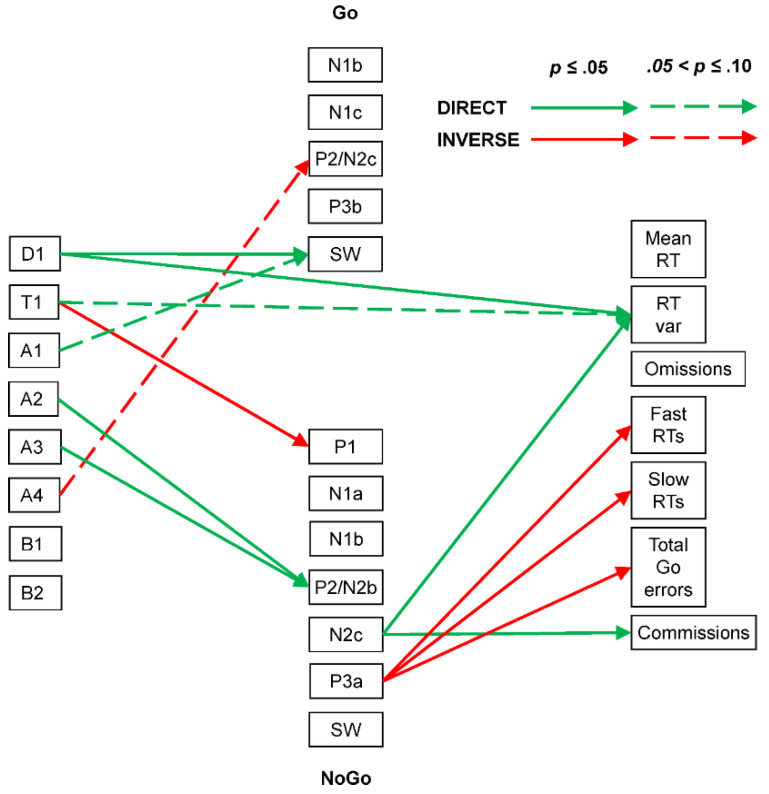
Young adult brain dynamics summary linking prestimulus frequency components (**left**), ERP components (centre: Go above NoGo), and behavioural measures (**right**). Note the simpler summary than that for the child group.

In terms of EEG–behaviour links, Go RT variability was directly affected by D1 (ρ = 0.47, *p* = 0.04, CI = [0.01, 0.77]) and somewhat also by T1 (ρ = 0.40, *p* = 0.08, CI = [−0.07, 0.73]). While none of the Go behavioural measures showed associations with the Go ERP components, NoGo commission errors were directly linked to NoGo N2c (ρ = −0.45, *p* = 0.05, CI = [−0.76, 0.01]). Go RT variability was directly associated with NoGo N2c (ρ = −0.58, *p* = 0.01, CI = [−0.82, −0.14]), and inverse associations were seen between NoGo P3a positivity and Go fast RT errors (ρ = −0.45, *p* = 0.05, CI = [−0.75, 0.02]), Go slow RT errors (ρ = −0.59, *p* = 0.01, CI = [−0.83, −0.16]), and Go total errors (ρ = −0.46, *p* = 0.04, CI = [−0.76, 0.00]). These links are shown in Figure 7.

### 3.3. Older Group

The older group averaged 3.4% NoGo commission errors and a total of 8.2% Go errors, comprising 2.2% Go omission errors, 0.1% fast RT errors, and 5.9% slow RT errors. Their mean RT was 366 ms (*SD* = 38), with a RT variability of 83 ms (*SD* = 11).

**Figure 8:**
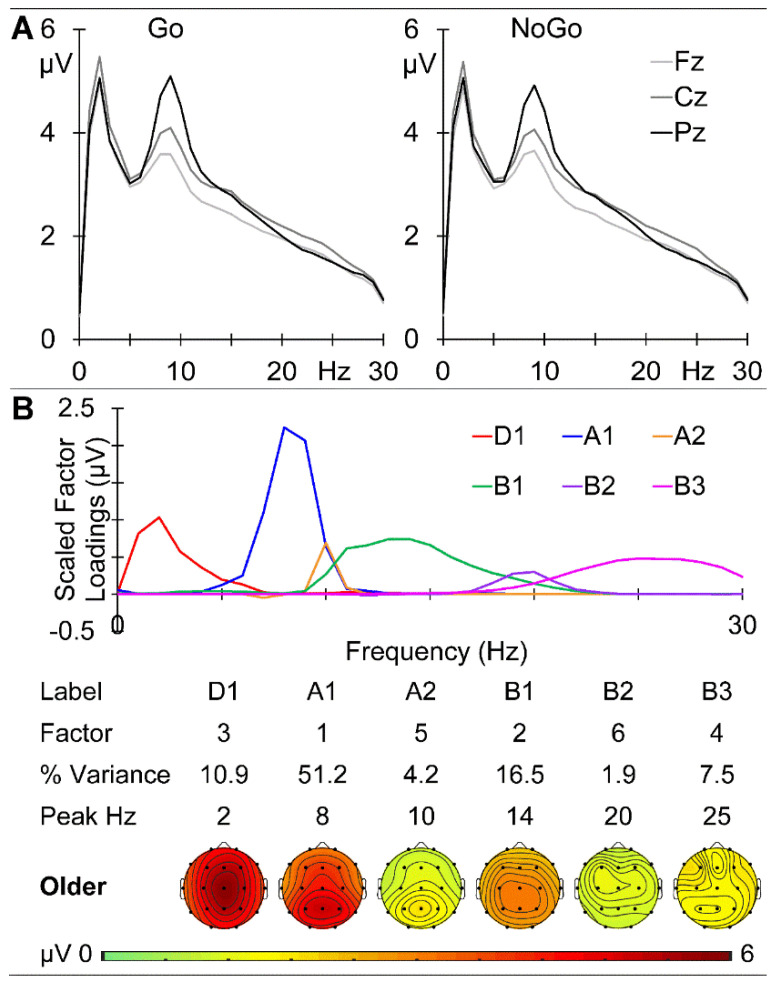
Older adult prestimulus spectra. (**A**) Prestimulus amplitude spectra at the midline sites before Go (**left**) and NoGo (**right**). (**B**) f-PCA outputs with the scaled factor loadings above the components in temporal order, together with their % variance, peak frequency and topographic headmaps.

The GM older group prestimulus EEG spectra at the midline sites are shown in Figure 8A. There is a clear peak at 2 Hz in the delta band and a marked parietal peak at 9 Hz in the alpha band, with activity declining over the beta range. The prestimulus activities before Go and NoGo stimuli were very similar, with the absolute maximum amplitude difference across frequency and sites being 0.175 μV. A combined f-PCA yielded one delta component (D1 at 2 Hz), two alpha components (A1 at 8 Hz and A2 at 10 Hz), and three beta components (B1 at 14 Hz, B2 at 20 Hz, and B3 at 25 Hz), each carrying at least 1.5% variance. Together these carried 90.3% of the spectral variance. Their scaled factor loadings, component information, and topographical headmaps are shown in Figure 8B.

**Figure 9:**
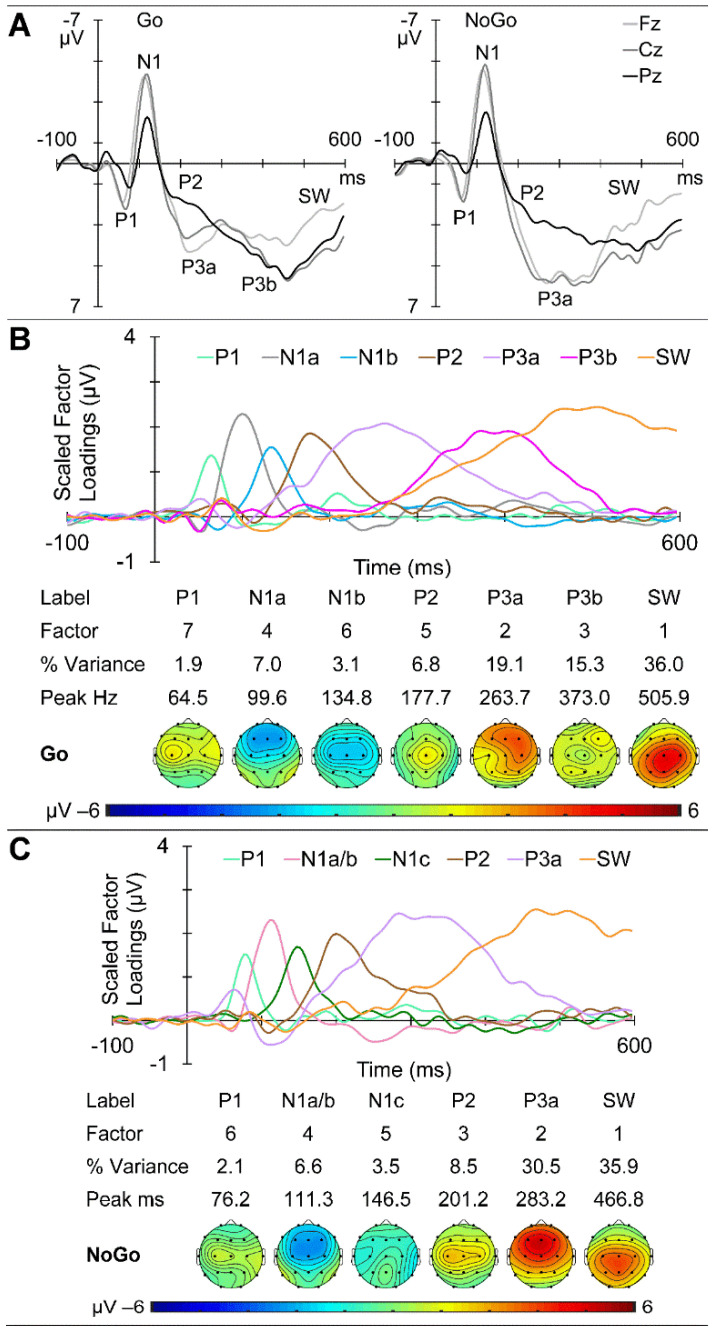
Older adult ERP results. (**A**) Go and NoGo response morphology at the midline sites as a function of time from stimulus onset. (**B**) t-PCA outcomes for Go: scaled factor loadings above the component information and component headmaps. (**C**) Results for NoGo components.

GM ERPs from the older group are shown in Figure 9A, with Go ERPs at the midline sites on the left and NoGo on the right. Clear P1 and N1 components are apparent in both sets, with subsequent morphological Go/NoGo differences apparent from approximately 250 ms. The Go ERPs yielded seven components carrying more than 1.5% variance each, for a total of 89.2% of the ERP variance. These were identified as P1, N1a, and N1b, followed by P2, P3a, P3b, and SW. Their scaled factor loadings and topographic headmaps are shown in Figure 9B. The NoGo ERPs yielded six components carrying more than 1.5% variance each, for a total of 87.1% of the total variance. These were identified as P1, N1a/b, and N1c, followed by P2, P3a, and SW. Their scaled factor loadings, component information, and topographic headmaps are shown in Figure 9C.

#### Older Global Amplitude Correlation Analyses

Spearman correlations in the older group indicated that Go N1a negativity was directly affected somewhat by A2 (ρ = −0.42, *p* = 0.07, CI = [−0.74, 0.05]) and significantly by B1 (ρ = −0.57, *p* = 0.01, CI = [−0.82, −0.13]), while Go P3b positivity was somewhat inversely affected by D1 (ρ = −0.42, *p* = 0.07, CI = [−0.74, 0.05]). NoGo P1 positivity was inversely affected by A1 (ρ = −0.51, *p* = 0.02, CI = [−0.79, −0.06]), A2 (ρ = −0.45, *p* = 0.05, CI = [−0.75, 0.01]), B1 (ρ = −0.48, *p* = 0.04, CI = [−0.77, −0.02]), and somewhat by B2 (ρ = −0.39, *p* = 0.09, CI = [−0.72, 0.08]). NoGo N1a/b negativity was directly affected somewhat by A2 (ρ = −0.40, *p* = 0.08, CI = [−0.72, 0.07]) and significantly by B1 (ρ = −0.47, *p* = 0.04, CI = [−0.77, −0.01]). NoGo SW positivity was somewhat inversely affected by A2 (ρ = −0.39, *p* = 0.09, CI = [−0.72, 0.08]) and B1 (ρ = −0.44, *p* = 0.06, CI = [−0.75, 0.03]).

In the only older group’s EEG–behaviour link, Go RT variability was directly affected by B3 (ρ = 0.51, *p* = 0.02, CI = [0.06, 0.79]). 

**Figure 10:**
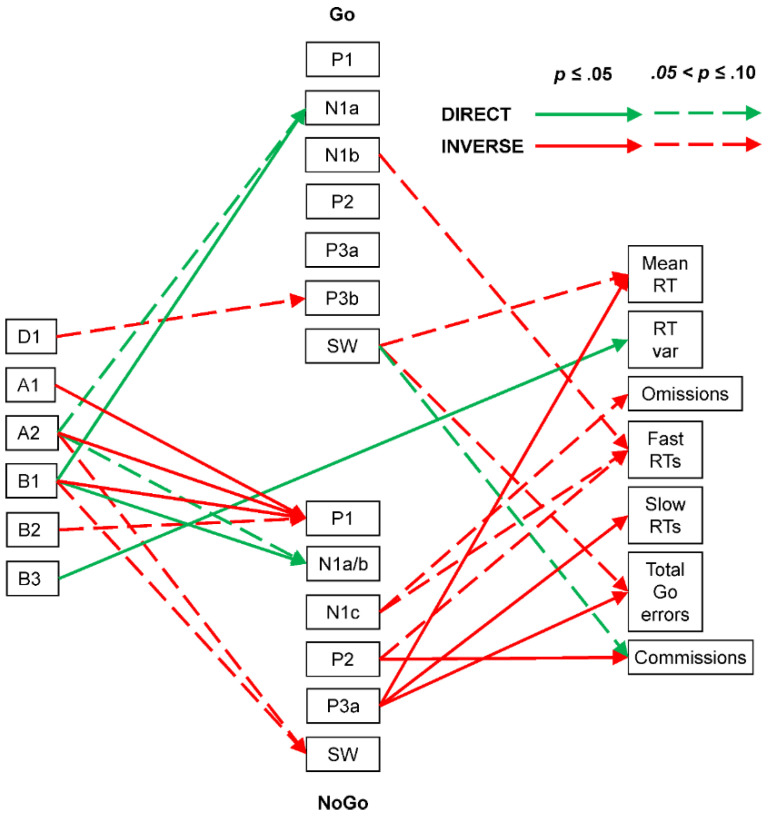
Older adult brain dynamics summary linking prestimulus frequency components (**left**), ERP components (centre: Go above NoGo), and behavioural measures (**right**). Note the increase in complexity compared with that in the young group’s summary.

In terms of ERP–behaviour links, near-significant inverse associations were seen between the Go SW positivity and both Go mean RT (ρ = −0.42, *p* = 0.06, CI = [−0.74, 0.05]) and total Go errors (ρ = −0.38, *p* = 0.10, CI = [−0.71, 0.09]) and between Go fast RT errors and Go N1b negativity (ρ = 0.38, *p* = 0.06, CI = [−0.09, 0.71]). NoGo commission errors were inversely linked to NoGo P2 positivity (ρ = −0.52, *p* = 0.02, CI = [−0.79, −0.07]) and showed some direct association with Go SW positivity (ρ = 0.37, *p* = 0.10, CI = [−0.10, 0.71]). Go mean RT was inversely associated with NoGo P3a (ρ = −0.53, *p* = 0.02, CI = [−0.80, −0.08]), while Go omissions were somewhat inversely associated with NoGo N1c negativity (ρ = 0.42, *p* = 0.06, CI = [−0.05, 0.74]), and Go fast RT errors were somewhat inversely associated with NoGo N1c negativity (ρ = 0.38, *p* = 0.10, CI = [−0.09, 0.71]) and NoGo P2 positivity (ρ = −0.38, *p* = 0.10, CI = [−0.71, 0.09]). Go slow RT errors and Go total errors were each inversely associated with NoGo P3a (each ρ = −0.71, *p* = 0.01, CI = [−0.89, −0.34]). These links are shown in Figure 10.

## 4. Discussion

This was an exploratory developmental study focussed on the brain dynamics involved in a fixed-interstimulus interval equiprobable auditory Go/NoGo task. We obtained novel prestimulus EEG measures *during* the task by using f-PCA to decompose EEG spectra obtained from the 500 ms before each stimulus onset, uncontaminated by poststimulus responses (i.e., ERPs or behaviour). t-PCA was then used to decompose the separate Go and NoGo ERP waveforms. Spearman correlations sought links between the prestimulus EEG components, ERP components, and behavioural measures. These analyses were carried out in separate groups of children, young adults, and older adults.

The sequential processing schema shown in Figure 1 was developed to help conceptualise the processing stages involved in this equiprobable auditory Go/NoGo task, and their links to ERP markers. The schema first covers the initial stages of sensory processing, during which the stimulus comes to be categorised as Go or NoGo. Subsequently, the processing splits to some extent, with the processing of the Go stimulus aimed at rapid response production and execution, and the NoGo processing aimed at inhibiting any such active response. Both these chains are followed by an evaluation of the performance in relation to the stimulus categorisation and preparation for the next stimulus. We remind readers that these stages are the simplified component peaks or “highlights” of the *parallel processing* indicated by the overlapping timing of the scaled ERP factor loadings plotted as amplitude vs. time (e.g., see Figure 3B,C). The ERP components found in each of our groups were broadly compatible with the component stages in Figure 1 and our previous reports on children [4,6], young adults [31], and older adults [1]. However, the schema has developed over the years, and previous reports have been based on differing and developing EEG/ERP processing steps and PCA techniques. The current three-group data sets have been processed using the same EEG/ERP pipeline for comparability, and our current optimised f-PCA and t-PCA techniques, allowing a coherent examination of developmental brain dynamics across the lifespan.

### 4.1. Changes with Age

#### 4.1.1. EEG

The EEG data examined here were extracted from 500 ms immediately prestimulus in an equiprobable Go/NoGo task and are relatively novel in reflecting task rather than rest conditions. The dominant low-frequency activity (primarily delta) was frontal in the children and young adults (see Figure 2 and Figure 5) but central in the older adults (Figure 8); delta amplitude reduced from some 11 to 7 to 5 µV with age. In contrast, the 9 Hz alpha peak remained parietal but reduced from approximately 7 to 6 to 5 µV over this age range, while beta showed little change over age. This systematic age reduction across the spectrum is compatible with resting-state expectations [13,16] and reflects the expected greater reduction in slow than fast oscillations. The f-PCA components broadly reflected these changes.

#### 4.1.2. ERPs

The general ERP morphology was similar across the age groups, with P1 followed by the N1, P2, N2, P3, and SW components; NoGo showed a larger central P3a compared with parietal Go P3b. The major stand-out is the extremely large frontal N2 apparent in children, which appears larger in NoGo than Go. The parietal/central Go/NoGo P3b/P3a differences appear to reduce with age. The t-PCA results largely reflected these expected components and matched previous age-related components (e.g., in children and young adults [5] and in young and older adults [1]).

#### 4.1.3. Behaviour

As expected, children performed relatively poorly in the task compared with the young adults, and the older adults showed some decline in performance (see [1,4]). The young adult sample showed faster mean RT and greater consistency in their responding (as indicated by their lower RT variability) relative to the child and older adult samples. This U-shaped pattern in response speed is typical of the developmental and ageing literatures [34,35] and may be attributable to the implementation of multiple strategies in this task and/or changes in brain morphology [35].

### 4.2. Brain Dynamics

#### 4.2.1. Child

Behaviourally, the child group had the slowest RTs; greatest RT variability; and highest number of Go omissions, fast RT errors, slow RT errors, total RT Go errors, and NoGo commission errors. These data confirm our previous results that children find this task difficult and require active inhibition when processing the NoGo stimulus [6], which is most apparent in their large dominant N2b ERP component (see Figure 3).

The summary of our child findings (Figure 4) indicates that this difficulty is reflected in a complex array of EEG/ERP/behaviour links. The child Go sensory processing ERP components reflect both facilitation and inhibition links with delta, alpha, and beta components, where corresponding NoGo sensory components are somewhat facilitated only by beta. Go and NoGo categorisation component markers are also substantially different in their links to EEG components, with only the Go N1c/P2 positivity being linked to delta, alpha, and beta activity. In the subsequent Go processing chain, N2b is unexpected, given its traditional link with P3a, which was not large enough in the Go chain to be extracted here. Go P3b was significantly reduced by delta activity, while delta and beta activity directly facilitated the evaluatory Go SW. In the NoGo processing chain, the inhibitory marker N2b was inversely affected by alpha; the unexpected N2c/P3b components were, respectively, increased by beta and higher delta, perhaps reflecting similar impacts of higher and lower levels of attention/vigilance. NoGo SW was enhanced by higher delta and reduced by lower delta, perhaps suggesting a link between outcome evaluation, the innervation of the unexpected N2c and P3b, and lower levels of attention/vigilance.

There were many apparent links between the Go and NoGo ERP components and behaviour. The most important of these in Go linked P3b inversely with omissions, RT variability, and slow RT errors, illustrating the expected role of P3b in this condition of the task. Moreover, the unexpected NoGo P3b showed some direct association with NoGo commission errors, reiterating the link between P3b and response execution (both valid as in Go and invalid as in NoGo). The unexpected NoGo N2c linked directly to fast RT errors, pointing to the non-functional role of this component, along with P3b, in child NoGo processing. The N2b component showed (near significant) direct associations with Go RT variability *irrespective* of its stimulus condition; this component is typically seen in the NoGo processing stream in conjunction with P3a, and its links here with RT variability likely reflect immature development of these stimulus-specific linkages. Surprisingly, NoGo P3a was associated with reduced mean RT, as well as omission, slow RT, and total Go errors.

Interestingly, prestimulus EEG components themselves were also linked to behavioural outcomes in children. High delta was directly linked with Go omissions and more so with NoGo commission errors, suggestive of the effects of reduced attention on the task, while high beta was also directly linked to NoGo commission errors, perhaps suggestive of over-activation and reduced inhibitory control.

#### 4.2.2. Young Adult

The young group had the best behavioural performance, with the shortest RT, smallest RT variability, fewest Go omissions, fewest slow RT errors, fewest total Go errors, and fewest NoGo commission errors.

The summary of their EEG/ERP/behaviour links (Figure 7) is also the simplest, suggesting an efficient implementation of brain dynamics in this paradigm. Only their theta activity affected sensory processing, with an inhibitory link between T1 and NoGo P1. Alpha (A4) inversely affected Go P2/N2c and facilitated (A2 and A3) NoGo P2/N2b, suggesting the role of focussed attention in the young categorisation of the Go/NoGo stimuli. In the NoGo processing chain, the unexpected N2c (the usual precursor of P3b) directly linked to more RT variability and commission errors, aspects of impaired NoGo processing. Surprisingly, NoGo P3a was linked to fewer fast and slow RT errors, and total Go errors.

Prestimulus EEG activity also impacted behavioural measures. Lower frequencies (D1 and T1) affected RT variability (i.e., with more low-frequency EEG, RT was more variable). This link may reflect the impact of lapses in attentional focus.

#### 4.2.3. Older Adult

The older group had somewhat poorer behavioural performance than the young group (mean RT, RT variability, omission errors, slow RT errors, and NoGo commission errors), suggesting a reduction in performance with increasing age in adults.

Their EEG/ERP/behaviour summary (Figure 10) also indicates more complex brain dynamics than in the young group. EEG–ERP links in the Go processing stream showed that A2 and B1 directly linked to both Go N1a and NoGo N1a/b, confirming older participants’ attention/vigilance involvement in the sensory-processing stage of the task. Alpha (A1 and A2) and beta (B1 and B2) prestimulus EEG components also inversely linked to NoGo P1, suggesting that similar EEG processes facilitating sensory processing in general reduced the earlier P1 stage in NoGo processing. The functional role of P1 in this paradigm is not yet well understood, but its reduction might facilitate greater N1a negativity (and associated processing) in the NoGo processing chain, perhaps due to the utilisation of a proactive (cf. reactive) cognitive control approach, or a wider mix of proactive and reactive control mechanisms across the task [20]. No prestimulus EEG components appear linked to the Go/NoGo categorisation markers in the older group, but Go P3b and NoGo SW appear inversely linked to D1 and A2/B1, respectively. This suggests that loss of attentional focus (D1) might impact Go responding (P3b), and attentive/vigilant states (A2/B1) might lead to better NoGo performance, not requiring extensive evaluation (SW).

Enhancement of Go sensory processing (N1b) was associated with fewer fast RT errors, while that of NoGo sensory processing (N1c) surprisingly appeared linked to reduced omissions and fewer fast RT errors. Go SW was linked with reduced mean RT and less total Go errors but, surprisingly, more commission errors. In the NoGo processing stream, the P2 categorisation marker was linked with reduced fast RTs and less commission errors, while the subsequent NoGo P3a was associated with lower mean RT and fewer slow RTs and total Go errors.

Only one prestimulus EEG component appeared linked to behavioural measures in the older group: high beta (B3) was directly linked to RT variability, suggesting that variation in high-activation EEG was associated with increased response variability. Here, the increased beta activity may reflect a reduction in dopaminergic function [36], which likely impacted cognitive motivation and the implementation of control strategies within this participant sample and task [37].

### 4.3. The Processing Schema across Ages

Our processing schema illustrated in Figure 1 generally accommodated the Go/NoGo ERP t-PCA outcomes from the three age groups and supported our expectations of similar sensory-processing and categorisation stages, leading to separate Go and NoGo processing stages and ending in similar evaluative processing, as reflected in the SW component. However, in all three groups, NoGo P3a amplitude was linked to improvements in Go behavioural measures, suggesting that this aspect should be included in future iterations of the schema. Perhaps P3a inhibition and the SW evaluation mark processing common to both Go and NoGo stimuli after the RT response has been activated or inhibited, respectively.

Deviations from the schema in the child group included Go N2b, and NoGo N2c and P3b, suggestive of poorly developed differentiation of Go versus NoGo processing and reflecting their greater difficulty and poorer performance in the task. In the older group, the appearance of Go P3a also deviated from expectations, although this has been found in some of our prior work [20]. This could reflect a “brake” on the RT production associated with the P3b and might reflect a strategic difference where the older group concentrated on accuracy rather than speed, as suggested in [1].

### 4.4. Limitations

Although our groups are matched on sex and handedness, they are relatively small (each *N* = 20), and this reduces confidence in the present results. However, our aim here was to present a preliminary study that could supply ideas and suggestions for future research in the developmental brain dynamics sphere. The novel EEG/ERP/behaviour summaries from each age group reflect achievement of our aim and provide many interesting linkages that should spur research on numerous fronts.

Another limitation is that the brief (50 ms duration) “beep” stimuli presented to the older group were 70 dB (cf. 60 dB) and included rise/fall times of 15 ms compared with the 5 ms used with the other groups. These changes were designed to ensure the clear audibility and differentiability of the Go and NoGo stimuli for all participants, recognising the link between age-related hearing decline and cognition [24,25]. The alternative to using identical stimuli could be explored in future studies but would require testing of hearing levels and may severely restrict the number of available older participants.

## 5. Conclusions

This exploratory study has shown developmental changes from children to younger adults and older adults in novel prestimulus EEG f-PCA components, ERP t-PCA components, and behaviour based on performance in an equiprobable auditory Go/NoGo task. Our framework was a t-PCA-based processing schema, which largely accommodated the ERP components and behavioural data, although the consistent unexpected connections between NoGo P3a and Go behaviours suggest the need for further development. Links between prestimulus EEG components, ERP components, and behaviour were obtained using simple Spearman correlations. These generated three age-specific summary plots that illustrate the wide range of brain dynamics involved in performing this task, and their variation throughout the lifespan.

In addition to indicating the value of the present approach, these summary plots suggest fruitful areas to explore in future studies. Such studies should include a revised processing schema better accommodating the NoGo P3a and its links to behavioural measures. More importantly, future studies should use larger samples than those available for this study in order to enhance our confidence in the links reported here. Perhaps further studies could vary age in large samples to better examine longitudinal aspects of development. Also, more-focussed studies examining portions of the EEG/ERP/behaviour ranges, such as focussing on part of the Go or NoGo processing chain, could be enlightening. Such limited studies could use the present data to generate hypotheses to be formally assessed with more powerful statistical techniques, such as multiple regression analysis. Although this exploratory study illustrates the value of obtaining extensive data and understanding from one paradigm, such as the auditory equiprobable Go/NoGo task used here, there would also be value in exploring this approach with different tasks. However, a stepwise approach, varying one parameter at a time (such as the interstimulus interval, the NoGo probability, or the stimulus modality) would help optimise understanding of the new data.

## Figures and Tables

**Table 1:** Participant group characteristics.

			Group	
Characteristic	Measure	Child	Young	Older
Sample size	*n*	20	20	20
Biological sex	Female:Male	15:5	15:5	15:5
Handedness	Right:Left	17:3	20:0	20:0
Age (years)	Range	8–12	18–24	59–74
	*M* (*SD*)	9.75 (1.33)	19.90 (1.62)	67.75 (4.45)

**Table 2:** Data characteristics for each participant group.

			Group	
Data		Child	Young	Older
Channel Interpolations	Range	0–3	0–3	0–3
	*M* (*SD*)	1.30 (1.08)	1.35 (1.04)	1.10 (1.02)
Assessed epochs in each condition	Range	66–134	79–149	81–146
	*M* (*SD*)	102.85 (18.10)	121.60 (16.31)	127.35 (17.85)

## Data Availability

The data presented in this study are available on request from the corresponding author.
